# Synovitis mediates cartilage outcomes during weight-loss in knee osteoarthritis – 4-year follow-up data from the Osteoarthritis Initiative

**DOI:** 10.1016/j.ocarto.2025.100653

**Published:** 2025-07-17

**Authors:** Virginie Kreutzinger, Katharina Ziegeler, Gabby B. Joseph, John A. Lynch, Zehra Akkaya, Nancy E. Lane, Charles E. McCulloch, Michael Nevitt, Thomas M. Link

**Affiliations:** a Department of Radiology and Biomedical Imaging, University of California, San Francisco, CA, USA; b Department of Radiology, Ankara University, Ankara, Turkey; c Department of Medicine and Center for Musculoskeletal Health, University of California, Davis, Sacramento, CA, USA; d Department of Epidemiology and Biostatistics, University of California, San Francisco, CA, USA

**Keywords:** Synovitis, Weight-loss, MRI, Osteoarthritis

## Abstract

**Objective:**

Weight loss can modify the progression of osteoarthritis (OA), and this may, in part, be achieved by decreased synovitis, a known accelerator of cartilage degeneration. The purpose of this study was to investigate whether change in synovitis mediates the beneficial effect of weight loss on longitudinal cartilage outcomes.

**Method:**

We analyzed right knees with baseline Kellgren & Lawrence grades 1–3 of 1153 obese and overweight participants of the Osteoarthritis Initiative (OAI) cohort with Whole Organ MRI Scores (WORMS) and semi-quantitative assessment of effusion synovitis and synovial proliferation scores form 3T MRIs at baseline and 48 months. There were 295 participants with weight-loss >5 ​% and 858 stable weight controls. Ordered logistic regression was used to assess the association of weight-loss status with concurrent changes in synovitis as well as cartilage WORMS scores; models were adjusted for age, gender, race, presence of radiographic OA, and abdominal circumference at baseline. A mediation analysis was used to determine whether change in overall cartilage degeneration was mediated by change in synovitis scores.

**Results:**

Individuals who lost weight had significantly lower odds for a higher grade on the scale assessing change in overall synovitis (OR 0.72; 95%CI 0.54, 0.95; p ​= ​0.018). Mediation analysis showed that slowing synovitis during weight loss had a small mediating effect on longitudinal cartilage outcomes.

**Conclusion:**

Decreased cartilage degeneration during weight loss was partially mediated by concurrent deceleration in synovitis, showing that decreasing systemic inflammation during weight-loss may not be mirrored in imaging markers of joint inflammation.

## 1 Introduction

Synovitis is increasingly recognized as a key driver in the pathophysiology of osteoarthritis (OA), particularly in the knee joint [1,2]. This inflammatory process not only contributes to joint pain and stiffness but also accelerates cartilage degeneration and structural joint damage [3]. The link between inflammation and OA has been recognized and studied for decades; as early as 1975, George Ehrlich proposed a possible connection between synovitis and OA progression [4]. Subsequent research has reinforced the pivotal role of synovial inflammation in the disease's development, highlighting it as a critical target for intervention [5]. Thus, despite sparse evidence for their clinical efficacy [6], non-steroidal anti-inflammatory drugs, or NSAIDs, are still among the most widely used pharmacological interventions in osteoarthritis. The success in applying more targeted immune-modulating drugs from other areas of rheumatology to OA-related synovitis has been limited to date [7,8], but the wide array of pharmacological agents continues to hold promise in certain patient populations [9]. Nevertheless, the slow pace of advancement in treatment development highlights the thus far limited understanding of the inflammatory processes involved in the synovitis of OA.

One potentially modifiable contributor to synovitis in OA is obesity [10,11]. The global obesity epidemic has paralleled a rise in OA incidence, underscoring the interplay between these conditions. Beyond increasing mechanical stress on load-bearing joints, obesity fuels chronic low-grade systemic inflammation through the production of pro-inflammatory cytokines by adipose tissue [12]. These cytokines, such as interleukins and tumor necrosis factor-alpha, contribute to the localized inflammatory milieu within the knee joint, exacerbating synovitis and accelerating the progression of OA. Connected to, yet separate from, obesity itself, the accumulation of subcutaneous fat has been connected to unfavorable outcomes in knee OA [13]. This dual impact—mechanical and inflammatory—makes obesity a particularly potent driver of knee OA, especially in an aging population where OA prevalence is already high. Consequently, weight loss has consistently emerged as one of the most effective lifestyle interventions for reducing OA symptoms and slowing disease progression [14]. Recent studies have demonstrated that improvements in synovitis, as visualized through imaging, often accompany reductions in body weight, suggesting a critical pathway through which weight loss exerts its benefits [15].

This study aimed to investigate the extent to which decreasing joint inflammation, as visualized on MRI, mediates the beneficial effects of weight loss on cartilage degeneration in overweight and obese individuals at risk for or with mild to moderate knee OA.

## 2 Methods

### 2.1 Participants

**Fig. 1:**
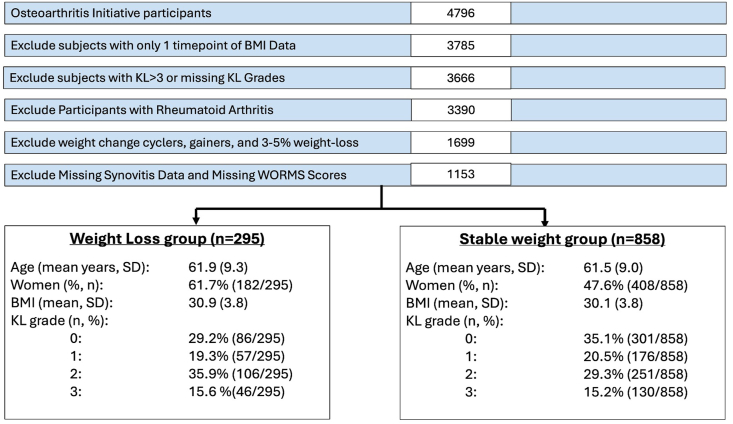
Flowchart of patient selection with exclusion criteria and demographics.

This study included participants from the Osteoarthritis Initiative (OAI), a multi-center, longitudinal observational study involving 4796 participants, aimed at assessing biomarkers in osteoarthritis. Information on demographics, weight status, synovitis, and MR imaging biomarkers of the right knee at study enrollment and 4-year follow-up were collected. We included participants with weight loss (n ​= ​295) and stable weight (n ​= ​858). Weight loss was defined as a reduction of baseline BMI >5 ​% in the 4-year follow-up period and stable weight as a BMI change of less than 3 ​% in either direction in the same period, following group definitions from previous work by Joseph et al. [16]. Exclusion criteria were history of reported rheumatoid arthritis or another inflammatory joint disease, BMI<25 ​kg/m^2^, incomplete BMI measurements or incomplete imaging data between baseline and 48 months, weight cyclers (recurrent loss and gain of weight, i.e. cyclic rather than linear weight change [16]) and participants with Kellgren & Lawrence grade 4 osteoarthritis in right knee radiographs. A flowchart detailing participant inclusion, along with demographic characteristics, is presented in Fig. 1. At each participating center, the institutional review boards approved the informed consent documents, study protocols, and any amendments. All research activities adhered to the principles of the Helsinki Declaration in its latest version and all data and materials presented in this study are available at OAI, https://nda.nih.gov/oai.

### 2.2 Image acquisition

All participants underwent a 3T MRI of the knee at baseline and again after a 4-year follow-up as part of the OAI, utilizing four identical MR scanners (Trio, Siemens Medical Solutions, Erlangen, Germany) across four different locations: University of Maryland School of Medicine, Baltimore, MD; the University of Pittsburgh, Pittsburgh, PA; the Ohio State University, Columbus, OH; and Memorial Hospital of Rhode Island, Pawtucket, RI. The MRI protocol of the knee, previously published [17], included a coronal 2D intermediate-weighted (IW) turbo spin-echo sequence, a sagittal fat-saturated 2D IW TSE sequence, and a sagittal 3D dual-echo steady-state (DESS WE) sequence with water excitation; these sequences were used for image analysis performed in this study.

### 2.3 Image analysis

#### 2.3.1 WORMS grading

We evaluated the MR images for OA features by using the established UCSF modified [18] whole organ MRI score (WORMS) [19]. Cartilage lesions were graded in 6 regions (lateral tibia, lateral femoral condyle, medial tibia, medial femoral condyle, trochlea and patella) using an eight-point scale. A score of 0 represented normal cartilage, and higher levels indicated increasing severity of cartilage lesions, with scores up to 6 denoting diffuse (>75 ​%) full-thickness loss in one compartment. We calculated a sum score for WORMS cartilage to represent the overall severity of cartilage damage, using this as the primary outcome measure. Bone marrow edema-like lesions (BMELLs) were evaluated individually in the patella, trochlea, medial femoral condyle, medial tibia, lateral femoral condyle, and lateral tibia. They were characterized as regions of increased T2 signal intensity and classified on a four-point scale from 0 (absent) to 3 (>20 ​mm in diameter). Subchondral cysts were assessed on a scale from 0 to 3 across the same six subregions of the knee joint, overlapping with the WORMS cartilage/BMELL regions. A score of 0 indicated no cysts, 1 represented cysts less than 3 ​mm in size, 2 corresponded to cysts measuring 3–5 ​mm, and 3 indicated cysts larger than 5 ​mm. For analysis, WORMS scorings were combined into sum scores, for the whole joint, and for the patellofemoral and femorotibial joint separately.

#### 2.3.2 Synovitis grading

To evaluate MR biomarkers of synovial inflammation we used two different scores: the MRI Osteoarthritis Knee Score (MOAKS) [20] and the synovial proliferation score (SPS) [21]. MOAKS was scored on reconstructed axial 3D DESS sequences using a 4-point scale ranging from 0 (normal physiological levels) to 3 (significant, causing capsular distention). The synovial proliferation score (SPS) included the following grades: 0 if no change in the synovial lining was observed, 1 if there was any synovial irregularity and 2 if there was extensive villonodular proliferation. Since a recent study [15] suggested that Hoffa synovitis may be a suboptimal marker for accurately grading synovial inflammation in weight losers, as an increased Hoffa signal score may reflect fat pad reduction rather than active synovitis, we focused exclusively on MOAKS and SPS for synovitis assessment in this cohort. To evaluate the overall level of synovitis, we calculated a sum score from both MOAKS and SPS (range per timepoint: 0 to 5; range for change: 5 to 5), which was used as the primary outcome measure for synovitis.

### 2.4 Statistical analysis

All analyses were performed using R Version 4.5.1 (packages: mediation, boot, emmeans, MASS, lmtest). We used ordinal logistic regression to estimate the adjusted odds of higher outcome scores between exposure groups, with covariate adjustment (sex, age, race, presence of radiographic OA (KL ​≥ ​2) at baseline, and abdominal circumference at baseline) and inference based on estimated marginal means and their contrasts. To assess the extent to which the beneficial effects of weight loss on structural imaging outcomes (WORMS) were mediated by change in synovitis, we performed a mediation analysis. This mediation analysis was based on counter-factual definitions of natural direct effects (NDE) and natural indirect effects (NIE) as proposed by Imai et al. [22]. Mediator and outcome models were fitted with ordinary least squares, treating both variables as continuous. NIE and NDE were estimated via non-parametric bootstrap with 1000 resamples [23]. The mediator (change in synovitis sum score) was regressed on the binary exposure (weight loss of ≥5 ​% over 4 years), covarying for sex, age, race, presence of radiographic OA (KL ​≥ ​2) at baseline, and abdominal circumference. The outcome model included the exposure, mediator and the same covariates. We obtained NIE, NDE and total effect (bootstrapped), reporting percentile-based 95%CIs. P-values <0.05 were considered statistically significant for all tests.

## 3 Results

### 3.1 Demographics

A total of 1153 participants were included in this analysis; of these, 295 had sustained weight loss of at least 5 ​% over 4 years, whereas 858 had stable weight (within 3 ​% of baseline BMI). A table of baseline demographics in both groups is integrated in Fig. 1. All participants had an elevated baseline BMI and age was similar in weight loss (61.9 ​*±* ​9.3 years) and stable weight (61.5 ​*±* ​9.0 years) participants. Differences between both groups were found in the proportion of women with 61.7 ​% (182/295) in the weight-loss groups vs. 47.6 ​% (408/858) in the stable weight group. Racial set up of groups differed significantly (p ​< ​0,001) with a higher proportion of Black or African American participants in the weight-loss group (25.7 ​% (76/295) vs. 16.2 ​% (139/858)).

### 3.2 Weight-loss and synovitis

**Fig. 2:**
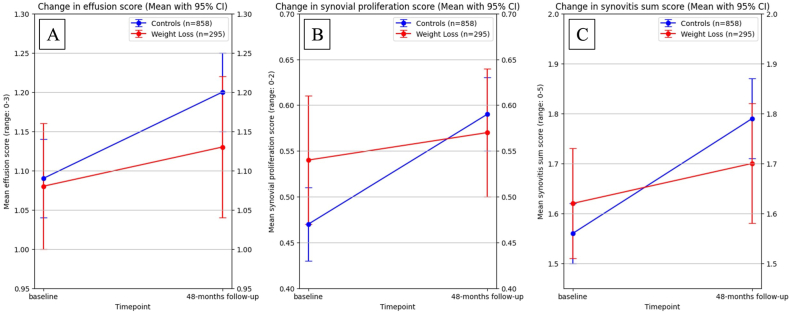
**Adjusted mean change in synovitis over 4 years.** Mean synovitis scores with change over 4 years. A: Effusion synovitis (difference in slope not statistically significant). B: synovial proliferation, significantly steeper slope in stable weight participants (blue line). C: Sum score of both markers, significantly steeper slope in stable weight participants (blue line). All analyses adjusted for age, sex, gender, race, baseline BMI, baseline abdominal circumference.

Individuals who lost weight had significantly lower odds for synovitis progression with an OR of 0.72 (95%CI 0.54, 0.95; p ​= ​0.018). When assessing two components of the synovitis score, we found this effect to be significant for synovial proliferation (OR 0.66, 95%CI 0.48, 0.91, p ​= ​0.013) but not for effusion synovitis (OR 0.82, 95%CI 0.61, 1.09, p ​= ​0.170). A graphical representation of adjusted mean synovitis scores is shown in Fig. 2.

### 3.3 Weight-loss and MR imaging outcomes of KOA

The results of ordered logistic regression analyses are listed in Table 1. A significant beneficial effect of weight-loss status on change in imaging biomarkers could be shown for cartilage lesion sum score with an OR of 0.76 (95%CI 0.59, 0.97; p ​= ​0.026). Neither change in overall BMELL nor subchondral cyst scores was significantly affected by weight-loss status, overall or in specific compartments.

**Table 1:** **Ordered logistic regression: weight-loss and structural imaging outcomes.** All regression analysis with weight-loss status (at least 5 ​% sustained weight-loss of 4 years) as the predictor and different WORMS outcomes. Analyses were adjusted for age, sex, gender, race, and baseline abdominal circumference.

Outcomes (WORMS)		OR	95 ​% Confidence interval		P value
			Lower bound	Upper bound	
Cartilage	**Overall**	**0.76**	**0.59**	**0.97**	**0.026**
	PFJ	0.81	0.62	1.05	0.104
	TFJ	0.84	0.65	1.08	0.172
BMELL	Overall	0.98	0.76	1.26	0.862
	PFJ	0.99	0.75	1.30	0.933
	TFJ	1.04	0.79	1.37	0.781
Subchondral cysts	Overall	0.82	0.61	1.09	0.170
	PFJ	0.88	0.64	1.22	0.448
	TFJ	0.77	0.53	1.10	0.145

### 3.4 Mediation of weight-loss effects by synovitis

**Fig. 3:**
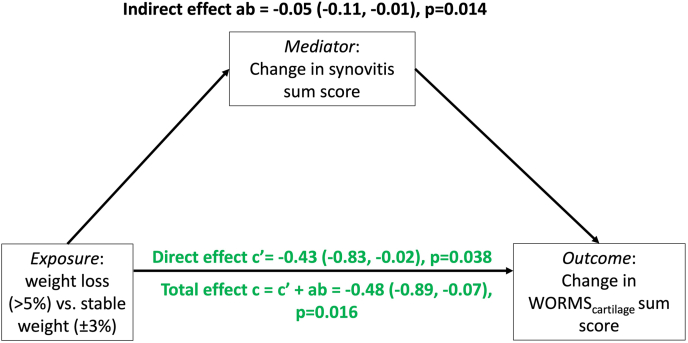
**Mediation of weight-loss effects on cartilage by synovitis.** Directed acyclic graph (DAG) of mediation analysis. All analyses adjusted for age, sex, gender, race, and baseline abdominal circumference.

A mediation analysis was performed for cartilage sum scores as the outcome, since change in all other structural imaging outcomes was not significantly affected by weight-loss status. A schematic of this mediation is provided in Fig. 3. This diagram shows that synovitis is positively associated with a change in cartilage outcomes, i.e., progression of cartilage damage. As weight loss leads to a decrease in synovitis, there is a small but significant indirect, or mediating, effect of synovitis in the interplay of weight loss and cartilage outcomes.

## 4 Discussion

Our investigation demonstrated that the beneficial effects of weight-loss on cartilage imaging outcomes in osteoarthritis can be partially attributed to decreased joint inflammation, visualized on MRI. While the mediating effect shown was modest, our results nevertheless have implications for the understanding of the interplay of systemic changes in body composition and local changes in joint tissues.

Our results demonstrated a significant effect of weight-loss on the progression of synovitis. It is noteworthy, however, that weight-loss participants still exhibited increases in synovitis over the observation period – just at slower rates than stable weight controls. Interestingly, the effects of weight loss were more pronounced for synovial proliferation than for effusion synovitis. Our findings indicate that weight loss alone does not stop the progression of synovitis, and further interventions targeting inflammation may potentially add benefit. Our results add to previous research that showed a mediating effect of worsening effusion synovitis on the association between BMI and radiographic progression in obese individuals [24]; together, these findings further establish the links between metabolic state, knee inflammation, and structural OA progression. Our results carry potential clinical significance in light of the observation that obese OA patients taking GLP-1 agonists like semaglutide experience a reduction in knee pain [25], which is associated with synovitis on MRI [26].

When interpreting the small magnitude of the effect we were able to show, it is essential to acknowledge that the imaging markers to quantify synovitis employed in our study, while well-established in osteoarthritis (OA) research, come with notable limitations. One major consideration is the dynamic [27] nature of synovitis in OA. Unlike cartilage degeneration, which typically progresses in a unidirectional and worsening manner over time, synovitis often exhibits a more fluctuating course. While there are cases where synovitis may worsen progressively, it is generally regarded as an OA feature that can increase or decrease over time, similar to bone marrow edema-like lesions. These lesions can be evident during baseline imaging but may completely resolve in subsequent follow-up scans. This inherent variability poses challenges in measuring synovitis reliably. One key issue in synovitis assessment arises from the imaging modalities available. Conventional, non-enhanced MRI is effective for detecting inflammation when effusion is present, as effusion serves as a reliable surrogate marker for synovitis. However, in the absence of effusion, identifying synovitis becomes significantly more challenging. In such cases, contrast-enhanced MRI (CE-MRI) is considerably more sensitive and effective, as supported by numerous studies [28,29]. Over the last decade there have been a number of additional technical advances in the depiction of synovitis, including contrast-free MRI techniques such as double inversion recovery [30] which offers the capability to enhance visualization of the synovium without the need for contrast agents by simultaneously suppressing signals from fluid and fat that may be utilized in future trials.

The OAI, from which we selected our study cohort, relies on non-contrast-enhanced MRI which limits the precision of synovitis assessment. Apart from the imaging technique, the semi-quantitative methods used have limited sensitivity to longitudinal change. There are multiple scoring systems for evaluating synovitis, each with distinct advantages and drawbacks. For instance, the MOAKS (MRI Osteoarthritis Knee Score) [20] effusion-synovitis metric and the ACLOAS (Anatomic Correlates of Lesions in OA Studies) [31] effusion-synovitis metric essentially measure the same feature but differ in their imaging planes—axial versus sagittal. A widely accepted approach involves measuring synovial proliferation [3], but this may be challenging to assess in the absence of joint effusion. Common to all currently used systems is the semi-quantitative assessment, which can fail to capture subtle longitudinal changes. Advances in tissue segmentation may allow for a more quantitative approach to measuring effusion/synovitis, allowing for higher levels of measurement precision. In our investigation, we deliberately chose not to include Hoffa's synovitis as a parameter. Recent findings have highlighted potential issues with this metric, particularly in patients experiencing weight loss [15]. In such cases, a decrease in the size of the fat pad could be misinterpreted as increased synovitis activity when this change is actually related to an increase in fluid due to a reduction in adipose tissue rather than active inflammation. This process is similar to the observed increases in radiodensity of fatty tissues on CT observed in weight-loss [32].

The current understanding of the relationship between cartilage lesions and synovitis proposes a bi-directional relationship [33]. While in early disease systemic inflammation from obesity may trigger synovitis, inflammatory reactions to cartilage debris in advanced cartilage damage may lead to sustained synovitis, regardless of changes in systemic inflammation. As our study was insufficiently powered to investigate interactions between KL-grade and weight loss for synovitis, we refrained from reporting such formal interaction analyses; further research is necessary to investigate whether such effects exist.

Even if our methods may have underestimated synovitis because of the limitations in available imaging, it is unlikely that weight-loss effects are fully mediated by synovitis, or global inflammation. Rather, the decrease in weight leads to mechanical unloading, which may be beneficial [34]. It has been estimated that every pound lost translates to a 4-pound reduction in compressive forces acting on the knee during walking [35].

The definition of weight-loss in our study, like in many studies before, has relied on reductions in BMI. This narrow focus has recently been called into question [36] by the American Medical Association (AMA) as they no longer recommend the use of BMI alone when assessing patients with obesity. The AMA thinks that BMI alone does not adequately capture different dimensions of body composition, such as the relative proportions of subcutaneous fat, visceral fat, and muscle mass. While an increase in muscle mass without loss of fat would result in an overall weight gain, this may not be related to increasing systemic inflammation in the same way an increase in fat tissue would. Future research should include more comprehensive, multi-dimensional approaches to assessing body composition, which could provide deeper insights into the relationship between weight loss and synovitis.

Our study has additional methodical limitations that should be considered. The number of individuals that achieved a sustained weight-loss, even a modest one of just 5 ​% of baseline BMI (which for example would translate to a weight reduction of 4.5 ​kg in an individual weighing 90 ​kg at a height of 170 ​cm at baseline) was limited, which may have led to a lack of statistical power for more elaborate subgroup analyses. Owing to the semi-quantitative nature of our imaging outcome metrics, which necessitated ordered logistic regression, the interpretability of our estimates of change was somewhat limited, and informative more for directionality than magnitude of observed effects. Furthermore, using discontinuous categories of weight loss, rather than change in BMI as a continuous variable lead to the exclusion of individuals who exhibited 3–5 ​% weight loss, which in turn may have introduced a selection bias in this analysis. Additionally, the observational nature of our data inherently limits the ability to demonstrate causative effects, and the findings should not be interpreted with the same rigor as results from randomized controlled trials.

In conclusion, sustained weight loss has a beneficial effect on synovitis and cartilage, but not on BMELLs or subchondral cysts. The decrease of synovitis partially mediates the slowing of cartilage degradation. Further research applying both more refined, quantitative measures of joint inflammation and a multi-dimensional approach to the concept of “weight-loss” could elucidate differences of these effects at different stages of OA to inform targeted treatment or prevention strategies.

## Author contributions

The authors have made substantial contributions to the following sections: Conception and design (VK, KZ, GBJ, ZA, NEL, CEM, MCN, TML). Analysis and interpretation of the data (VK, KZ, GBJ, JAL, ZA, NEL, CEM, MCN, TML). Collection and assembly of data (VK). Drafting of the article (VK, TML). Statistical expertise (GBJ, CEM, MCN). Critical revision of the article for important intellectual content (VK, KZ, GBJ, JAL, ZA, NEL, CEM, MCN, TML). Final approval of the article (VK, KZ, GBJ, JAL, ZA, NEL, CEM, MCN, TML). Virginie Kreutzinger (v.kreutzinger@gmail.com) takes responsibility for the integrity of the work as a whole from inception to the finished article.

## Role of the funding source

This study was funded by NIH R01-AR064771, NIH R01-AR078917 and R01-AG070647. The OAI is a public-private partnership comprised of five contracts (N01-AR-2–2258; N01-AR-2–2259; N01-AR-2–2260; N01-AR-2–2261; N01-AR-2–2262) funded by the National Institutes of Health, a branch of the Department of Health and Human Services and conducted by the OAI Study Investigators. Private funding partners include Merck Research Laboratories; Novartis Pharmaceuticals Corporation, GlaxoSmithKline; and Pfizer, Inc. Private sector funding for the OAI is managed by theHave we correctly interpreted the following funding source(s) and country names you cited in your article: Novartis Pharmaceuticals Corporation, United States; MRI, United States; OAI, United States; Pfizer, United States; Foundation for the National Institutes of Health, United States; NIH, United States; Merck, United States? Foundation for the National Institutes of Health.

## Declaration of competing interest

None.
